# Physiological and transcriptomic analysis of the effects of different selenium and potassium application concentrations on the growth and development of banana seedlings

**DOI:** 10.1186/s12870-026-09472-z

**Published:** 2026-07-30

**Authors:** Jieyun Liu, Jiayu Peng, Manrong Gao, Weihua Huang, Junniu Zhou, Qinglan Tian, Yingjun Zhang, Zuxiang Su, Yanyan Wu, Haifei Mou

**Affiliations:** Guangxi Academy of Agricultural Sciences, Nanning, 530007 China

**Keywords:** Banana, Physiological characteristics, Selenium-potassium combined application, Transcription

## Abstract

This study aims to investigate the effects of selenium on potassium absorption and accumulation, as well as the influence of varying concentrations of selenium-potassium combined applications on the growth of banana seedlings. A combined selenium-potassium application experiment was conducted on Guijiao No. 9 banana seedlings, using potassium chloride (KCl) as the potassium source and sodium selenate (Na₂SeO₄) as the selenium source. At 20 and 40 days post-treatment, measurements were taken for the fresh and dry weights, as well as the selenium and potassium contents in the roots, stems, and leaves. Additionally, leaf antioxidant enzyme activities, soluble protein content, and soluble sugar content were assessed. Under a constant KCl concentration, an increase in Na₂SeO₄ concentration initially led to an increase in fresh and dry weights of the roots, stems, and leaves, along with enhanced antioxidant enzyme activities, soluble protein, and soluble sugar contents at 20 days post-treatment, which subsequently decreased. The potassium contents in the roots and stems showed no significant variation at 20 days but displayed an initial increase followed by a decline at 40 days, while leaf potassium content remained largely unchanged. The selenium contents in all plant tissues exhibited an increasing trend. Transcriptome analysis identified 2,455, 3,530, 3,134, 2,541, 3,422, 1,101, 471, and 3,221 differentially expressed genes (DEGs) between the groups YT1/YCK1, YT2/YCK2, YCK1/YCK2, YT1/YT2, GT1/GCK1, GT2/GCK2, GCK1/GCK2, and GT1/GT2, respectively. A significant number of these DEGs were enriched in the plant hormone signal transduction pathway. Notably, 472 DEGs from the GT1/GT2 comparison may play a key role in promoting selenium and potassium absorption in banana seedlings. The combination of KCl at 400 mg·L⁻¹ and Na₂SeO₄ at 1.0 mg·L⁻¹ increased the biomass of roots, stems, and leaves, as well as in selenium and potassium contents, and enhanced antioxidant enzyme activities, which facilitated the growth of banana seedlings. Kyoto Encyclopedia of Genes and Genomes pathway analysis revealed that DEGs were significantly enriched in plant hormone signal transduction pathways. Significance: The findings provide theoretical support for further studies on the physiological and biochemical effects, as well as the yield and quality of field-cultivated banana.

**Supplementary Information:**

The online version contains supplementary material available at https://doi.org/10.1186/s12870-026-09472-z.

## Background

Banana (*Musa acuminata* AAA Cavendish) is a significant food and economic crop in the subtropical regions of southern China [1], predominantly cultivated in provinces such as Guangxi, Guangdong, Hainan, and Fujian [2]. It is highly valued by consumers due to its rich nutritional profile, including phenolic compounds, mineral elements, resistant starch, and dietary fiber. Additionally, it has been recognized for its potential in preventing cardiovascular diseases, lowering blood lipids, blood pressure, and blood sugar levels, as well as promoting intestinal regularity [3]. Increasing the yield of banana is vital for securing market supply, and with the rising living standards, there is a growing consumer demand for high-quality banana. Both selenium and potassium are essential for plant growth and development [4, 5], and adequate intake of these elements is crucial for human health [6, 7]. Therefore, the application of selenium and potassium may offer a viable strategy for enhancing the quality and characteristics of banana.

Potassium is an essential nutrient for plants [8], playing a vital role in photosynthesis, protein synthesis, and the synthesis and transport of carbohydrates [9]. The beneficial effects of potassium on both yield and quality have been documented in various crops, including *Oryza sativa* [10], *Zea mays* [11], *Solanum tuberosum* [12], *Saccharum officinarum* [13], *Cucumis sativus* [14], *Solanum lycopersicum* [15], *Fragaria × ananassa* [16], and *Gossypium spp.* [17]. The impact of potassium on the growth, yield, and quality of banana has also been explored. banana is often regarded as a “potassium-loving” crop. Potassium deficiency in banana seedlings disrupts normal photosynthesis, thereby hindering proper plant growth [18]. A study by Xu et al. [19] revealed that critical genes associated with potassium transport and uptake, such as HAK/KUP/KT system genes, AKT family genes, and CBL genes, may play a role in the response of banana to potassium deficiency. However, excessive potassium fertilization can negatively affect plant growth [20]. Research by Xie Rulin et al. [21] identified a critical threshold for potassium deficiency in banana at 228 mg/L, with an optimal potassium concentration ranging between 228 and 539 mg/L. Sathappan C T. et al. [22] demonstrated that foliar application of 1.5% potassium sulfate, administered twice at 15-day intervals following bunch emergence, combined with 550 g of potassium per plant, represents the optimal treatment for improving the yield and quality of banana. These findings suggest that various fertilization methods and potassium concentrations influence the yield and quality of banana, highlighting the need for further comprehensive analysis.

Selenium (Se) is an essential nutrient for humans and animals, and green plants serve as the primary source of selenium for human and animal intake [23]. At appropriate concentrations, selenium exerts significant effects on the growth, development, and physiological and biochemical processes of crops [24–26], fruits [27–29], and vegetables [30–32]. Additionally, selenium plays a key role in the growth and development of banana. Zhang et al. [33] demonstrated that foliar application of selenium fertilizer resulted in an increase in both yield and fruit selenium content in banana to a certain extent. Liu et al. [34]reported that the soil drenching of sodium selenate produced differential effects across various banana cultivars. However, it consistently promoted plant growth, enhanced individual plant yield, and increased Vitamin C content in the fruit. Eisa et al. [35] found that the combined application of selenium with glutamic acid and seaweed extract significantly enhanced vegetative growth, bunch weight, and the physical and chemical characteristics of the fruit. Liu et al. [36] further observed that selenium application not only increased the selenium content in fruit to meet selenium-enrichment standards but also substantially mitigated damage caused by soil-borne pathogens in banana by enhancing antioxidant enzyme activities and inhibiting fungal growth. Common selenium fertilizers mainly include selenates, selenites and organic selenium products such as selenoglutamate. Existing experiments have verified that under the same application rate in acidic soils, selenates exhibit a better biofortification effect on crops than selenites. Selenites tend to bind firmly to soil organic matter, clay minerals and metal oxides, which endows selenite fertilizers with a long-term efficacy and meanwhile easily leads to selenium accumulation in soil. In addition, crops generally show low absorption and utilization efficiency of organic selenium [37]. Therefore, examining the effects of selenium application on the growth and selenium accumulation of banana under varying soil types and ecological conditions is crucial for the development of selenium-enrichment cultivation techniques for this crop.

Elements of selenium and potassium have impacts on plant growth and development. Previous studies have demonstrated that selenium application not only enhances the yield and quality of banana, but also facilitates the absorption of both selenium and potassium in banana fruits [38]. However, research on the combined application of selenium and potassium, particularly in relation to the physiological and biochemical characteristics, yield, and quality of banana, remains limited. This study employed Guijiao No. 9 banana seedlings as experimental material, with the aim of elucidating the effects of varying selenium and potassium application concentrations on seedling growth. Through transcriptome sequencing, key signaling pathways related to the selenium-potassium response were identified, further enhancing the understanding of the mechanisms through which the combined application of selenium and potassium influences banana growth. This study is of significant importance for promoting the growth of banana seedlings, as well as ensuring optimal yield and quality.

## Materials and methods

### Materials

#### Plant materials

Banana seedlings of the Guijiao No. 9 variety were cultivated at the Guangxi Academy of Agricultural Sciences.

#### Material treatment

Banana seedlings were cultivated in hydroponic pots with a height of 21 cm, an upper diameter of 21 cm, and a lower diameter of 15 cm, with one plant per pot and 4.5 L of nutrient solution per pot. On April 24, 2023, tissue-cultured banana seedlings at the 5–6 leaf stage, exhibiting uniform growth, were selected. After root removal, the seedlings were cultured hydroponically in Hoagland’s nutrient solution for a duration of 20 days. Various nutrient solution treatments were applied on May 13, 2023. A modified, potassium-free Hoagland’s nutrient solution was used, with eight distinct treatments established (Table 1). Each treatment included 24 plants, and the nutrient solution was replaced every 7 days. Relevant data were collected at 20 and 40 days post-treatment.

**Table 1:** Experimental Treatments

Treatments	KCl(mg·L^− 1^)	Na_2_SeO_4_(mg·L^− 1^)
T1	400(normal potassium)	0
T2	400	0.5
T3	400	1.0
T4	400	2.0
T5	100(low potassium)	0
T6	100	0.5
T7	100	1.0
T8	100	2.0

The modified potassium-free Hoagland’s nutrient solution formula consisted of: urea 180 mg/L, magnesium sulfate 493 mg/L, ammonium phosphate 115 mg/L, calcium nitrate 492 mg/L, ferrous sulfate heptahydrate 13.9 mg/L, ethylenediaminetetraacetic acid disodium salt 18.6 mg/L, boric acid 2.5 mg/L, manganese sulfate 2.5 mg/L, zinc sulfate 0.5 mg/L, pH = 6.0

### Experimental methods

#### Physiological indicator determination

The fresh weight and dry weight of banana leaves, stems, and roots were measured, with 6 plants assayed per treatment. For the determination of potassium (K) and selenium (Se) contents, dry samples of leaves, stems, and roots from 2 plants were uniformly mixed respectively, and each measurement was performed in 3 biological replicates. Potassium content was determined using the flame atomic absorption spectrometry [39], while selenium content was measured via the hydride generation atomic fluorescence spectrometry [36]. The 3rd fully expanded leaf (counted from the top of the plant) was sampled to determine the contents of soluble protein, soluble sugar, and the activities of superoxide dismutase (SOD), catalase (CAT), and peroxidase (POD). Samples from 3 plants were uniformly mixed for each assay, with 3 biological replicates conducted. The contents of soluble sugar, CAT, POD, and SOD were determined using commercial kits: Plant Soluble Total Sugar Content Kit (Cat. No. JC0401-M), Catalase Kit (Cat. No. JC0103-S), Superoxide Dismutase Kit (Cat. No. JC0101-M), and Peroxidase Kit (Cat. No. JC0102-S), all purchased from Nanjing Jice Biological Technology Co., Ltd. The content of soluble protein was determined by the Coomassie Brilliant Blue G-250 method.

#### Transcriptome Sequencing and Analysis of banana

At 20 days after treatment, the newly emerging young leaves and roots of banana plants under treatments T1, T3, T5, and T7 were collected and sent to Shanghai Majorbio Biomedical Technology Co., Ltd. for transcriptome sequencing (Table 2). Total RNA was extracted from 24 samples, followed by cDNA library construction. Sequencing was performed on the Nova sequencing platform, and the obtained sequences were aligned against the banana reference genome (https://banana-genome-hub.southgreen.fr/node/50/338). All transcripts were annotated by mapping to six databases, including NR (https://www.ncbi.nlm.nih.gov/public/),Swiss-Prot(ftp://ftp.uniprot.org/pub/databases/uniprot/current_release/knowledgebase/complete/uniprot_sprot.fasta.gz), KEGG(http://www.genome.jp/kegg/), EggNOG (http://eggnogdb.embl.de/#/app/home), GO (http://www.geneontology.org/), and Pfam (http://rfam.janelia.org/).

**Table 2:** Overview of sample information for transcriptome sequencing

Sample Information	Replicate IDs	Group Name	
Leaves under T1 treatment	Y2-1-1, Y2-1-2, Y2-1-3		YCK1
Leaves under T3 treatment	Y2-3-1, Y2-3-2, Y2-3-3		YT1
Leaves under T5 treatment	Y2-5-1, Y2-5-2, Y2-5-3		YCK2
Leaves under T7 treatment	Y2-7-1, Y2-7-2, Y2-7-3		YT2
Roots under T1 treatment	G2-1-1, G2-1-2, G2-1-3		GCK1
Roots under T3 treatment	G2-3-1, G2-3-2, G2-3-3		GT1
Roots under T5 treatment	G2-5-1, G2-5-2, G2-5-3		GCK2
Roots under T7 treatment	G2-7-1, G2-7-2, G2-7-3		GT2

Based on the quantitative results of gene expression levels, DESeq 2 software was employed for differential expression analysis. The Benjamin-Hochberg method was applied to adjust the generated p-values, aiming to control the false discovery rate (FDR). Differentially expressed genes (DEGs) between the two groups were identified using the screening criteria of : |log2FC| ≥0 and adjusted p-value (padjust) < 0.05. Functional enrichment analysis of genes/transcripts in the gene set was performed, including Gene Ontology (GO) enrichment analysis and Kyoto Encyclopedia of Genes and Genomes (KEGG) pathway enrichment analysis. GO enrichment analysis of genes in the gene set was conducted using Goatools software to interpret the main GO functions of the genes. The Fisher’s exact test was used, and a GO term was considered significantly enriched when the adjusted p-value (padjust) < 0.05. KEGG PATHWAY enrichment analysis of genes/transcripts in the gene set was performed using an R script, following the same calculation principle as GO functional enrichment analysis. A KEGG pathway was regarded as significantly enriched when the adjusted p-value (padjust) < 0.05.

#### Validation by Quantitative Real-Time PCR (qRT-PCR)

To verify the reliability of RNA-Seq data, five genes (*Macma4-04-g37860*, *Macma4-06-g36370*, *Macma4-02-g16650*, *Macma4-07-g23970*, and *Macma4-03-g19210*) were selected from the transcriptome data of root samples under T5 and T7 treatments, and their relative expression levels were determined by qRT-PCR (the primer sequences of the selected genes are shown in Table 3). Total RNA (1 µg) from 6 samples was reverse-transcribed into cDNA using the HiScript Q RT SuperMix for qPCR (+ gDNA wiper) kit. The qRT-PCR reaction system was prepared according to the instructions of the ChamQ SYBR Color qPCR Master Mix (2X) kit (Table 4), and qRT-PCR detection was performed on an ABI 7300 Real-Time PCR System (Table 5).

**Table 3:** Primer sequences used for qRT- PCR

Number	Gene Name	Primer Name	Primer Sequence (5’→3’)	Product Length
1	*Macma4−04-g37860*	LJY−04-g37860-F2	CTGTTCTGCTGGATGGACTT	224
		LJY−04-g37860-R2	TTCTCCCGGTACACGAAA	
2	*Macma4−06-g36370*	LJY−06-g36370-F1	TGTTATAGGGATAAGGGATG	194
		LJY−06-g36370-R1	TCTTGGTTGGAGCGGATT	
3	*Macma4−02-g16650*	LJY−02-g16650-F2	ATGAGGTCGGCAAATTACGC	194
		LJY−02-g16650-R2	ATGCGCTGGTTGGCGTAG	
4	*Macma4−07-g23970*	LJY−07-g23970-F1	TATCTTCTGGAGGAGGAGGAAC	168
		LJY−07-g23970-R1	GTCAGGCCAAACAACATCG	
5	*Macma4−03-g19210*	LJY−03-g19210-F	CGAGTTCGCCACGCTCTA	230
		LJY−03-g19210-R	CCTTGAAGTCCACCATCCC	
6	actin (Reference Gene)	actinF	CGAGGCTCAATCAAAGA	379
		actinR	ACCAGCAAGGTCCAAAC	

**Table 4:** Reaction System

Reaction Component	Volume (µl)
2X ChamQ SYBR Color qPCR Master Mix	5
Forward primer (F) (5µM)	0.4
Reverse primer (R) (5µM)	0.4
50 X ROX Reference Dye 1	0.2
Template (cDNA)	1
ddH_2_O	3
Total	10

**Table 5:** Reaction Program

Thermal Cycler	Times and Temperatures			
	Initial Steps	Each of 40 cycles		
		Melt	Anneal	Extend
	HOLD	CYCLE		
ABI7300 Real-Time PCR System	5 min	5 s	30 s	40 s
	95℃	95℃	55℃	72℃

PCR reaction program: initial denaturation at 95℃ for 5 min, then 40 cycles of 95℃ for 5 s (denaturation), 55℃ for 30 s (annealing), and 72℃ for 40 s (extension)

### Data analysis

All experiments were performed in triplicate, and data are presented as mean ± standard deviation, different letters indicate significant differences according to Duncan’s multiple range test at *p* < 0.05 using a one-way ANOVA. Microsoft Excel 2019, IBM SPSS Statistics 27, Prism, and TBtools were employed for data processing and graphical visualization. The relative expression levels were calculated by the 2^−ΔΔCt^ method to determine target gene copy numbers, which were normalized against reference genes.

## Results

### Effect of combined application of selenium and potassium on growth and physiological parameters of banana

#### Effects of combined application of selenium-potassium on banana biomass

At 40 days post-treatment, the fresh and dry weights of roots, stems, and leaves were higher than those observed at 20 days post-treatment, and the biomass of treatments T1-T4 exceeded that of treatments T5-T8. When the potassium chloride (KCl) concentration was 400 mg·L⁻¹, no significant differences were observed in the fresh and dry weights of roots, stems, and leaves of banana across different Na₂SeO₄ concentrations at 40 days post-treatment. However, at 20 days post-treatment, significant differences were observed in the fresh and dry weights of roots, stems, and leaves among the different Na₂SeO₄ concentrations. At a Na₂SeO₄ concentration of 1.0 mg·L⁻¹, the fresh weights of the roots, stems, and leaves reached their maximum values of 11.89 g, 38.29 g, and 39.86 g, respectively, representing 1.20, 1.12, and 1.43 times higher than the values at 0 mg·L⁻¹ Na₂SeO₄. The dry weights of the stems and leaves were 1.28 g and 3.44 g, respectively, which were 1.16 and 1.42 times of those observed at 0 mg·L⁻¹ Na₂SeO₄.

**Fig. 1:**
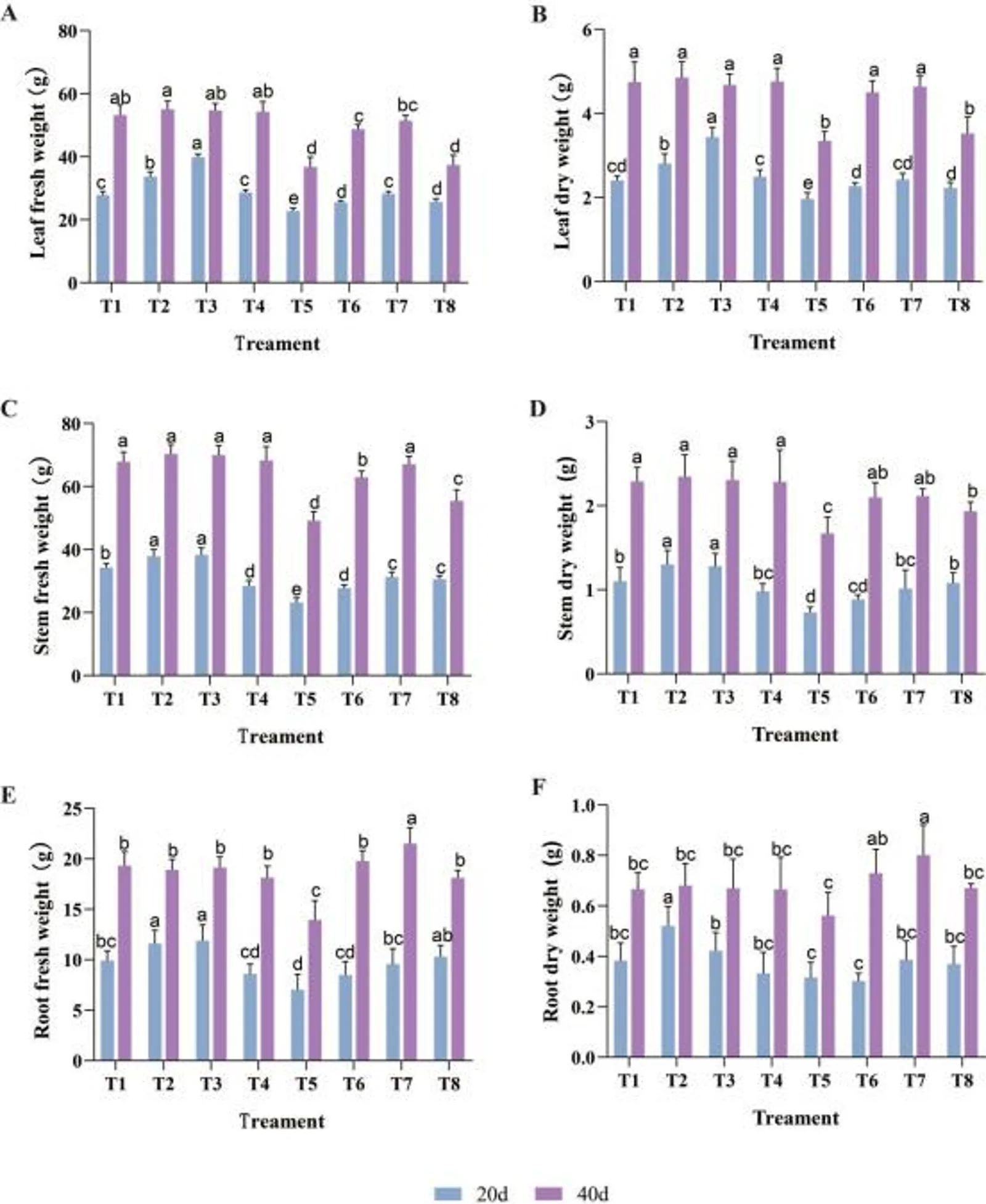
Biomass accumulation in different organs of banana seedlings under various treatments. **A**, Fresh weight of leaves; **B**, Dry weight of leaves; **C**, Fresh weight of stems; **D**, Dry weight of stems; **E**, Fresh weight of roots; **F**, Dry weight of roots

When the KCl concentration was 100 mg·L⁻¹, significant differences in the fresh and dry weights of roots, stems, and leaves of banana were noted across the different Na₂SeO₄ concentrations, with the biomass reaching its maximum values at a Na₂SeO₄ concentration of 1.0 mg·L⁻¹. At 20 days post-treatment, when the KCl concentration was 100 mg·L⁻¹ and Na₂SeO₄ concentration was 1.0 mg·L⁻¹, the fresh weights of the roots, stems, and leaves were 9.58 g, 31.27 g, and 28.26 g, respectively, representing 1.36, 1.35, and 1.24 times as those observed at 0 mg·L⁻¹ Na₂SeO₄. The dry weights were 0.38 g, 1.02 g, and 2.42 g, respectively, which were 1.22, 1.39, and 1.24 times of the values at 0 mg·L⁻¹ Na₂SeO₄. At 40 days post-treatment, the fresh weights of the roots, stems, and leaves were 21.52 g, 67.07 g, and 51.41 g, respectively, representing 1.54, 1.36, and 1.40 times higher than those at 0 mg·L⁻¹ Na₂SeO₄. The dry weights were 0.80 g, 2.12 g, and 4.64 g, respectively, which were 1.42, 1.27, and 1.39 times those at 0 mg·L⁻¹ Na₂SeO₄ (Fig. 1). These results indicate that, under low potassium treatment (KCl concentration of 100 mg·L⁻¹), varying Na₂SeO₄ concentrations exerted more pronounced effects on the roots of banana, with Na₂SeO₄ concentration of 1.0 mg·L⁻¹ leading to significant increases.

#### Effects of selenium-potassium combined application on antioxidant enzyme activities in banana leaves

**Fig. 2:**
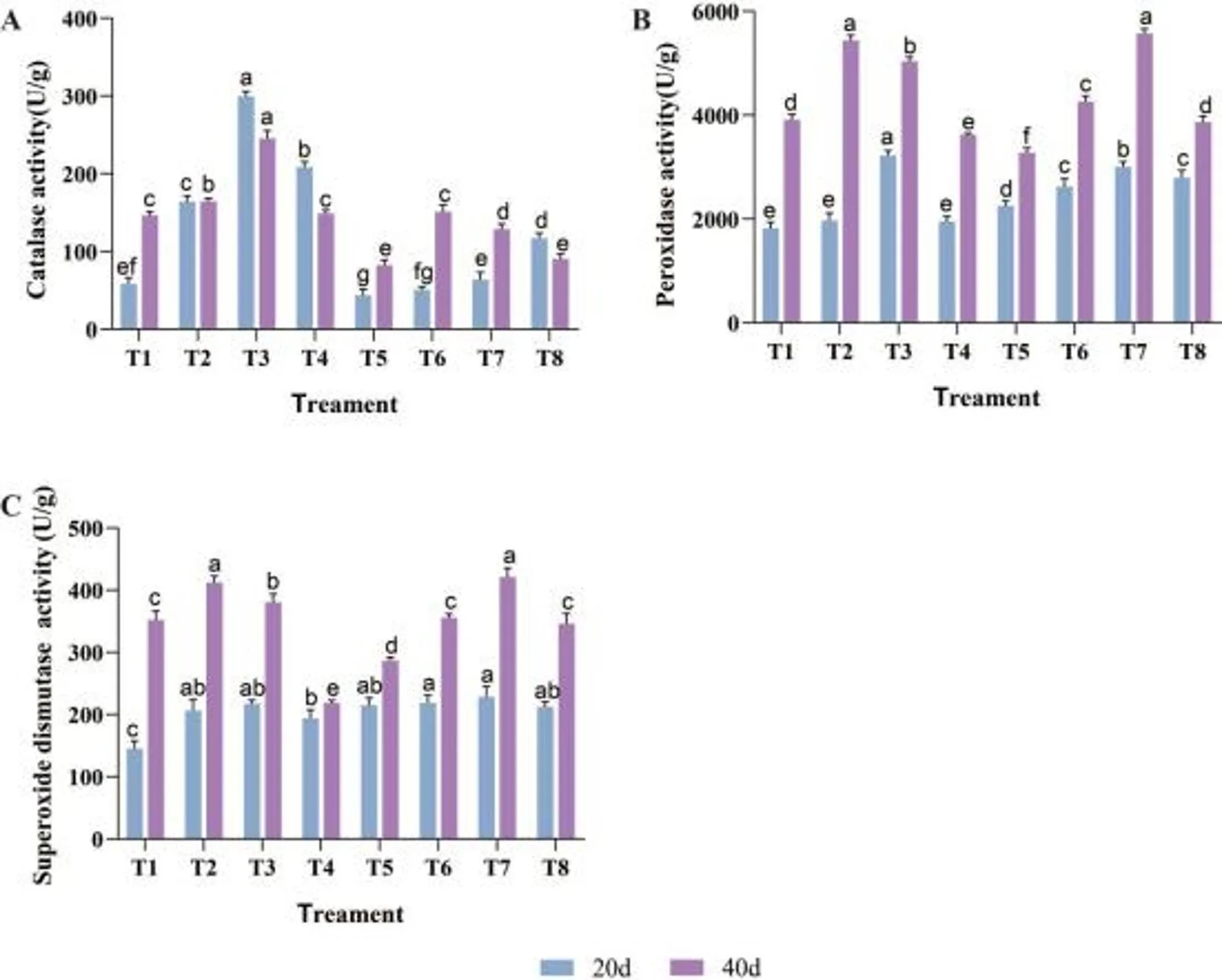
Activities of antioxidant enzymes in banana leaves under different treatments. **A**, Catalase activity; **B**, Peroxidase activity; **C**, Superoxide dismutase activity

When the KCl concentration was 400 mg·L⁻¹ and the Na₂SeO₄ concentration was 1 mg·L⁻¹, the highest catalase (CAT) activities at 20 and 40 days post-treatment were 299.41 U/g and 245.41 U/g, respectively, representing 5.13-fold and 1.68-fold increases compared to those at 0 mg·L⁻¹ Na₂SeO₄. The CAT activities in treatments T1-T4 were consistently higher than those in T5-T8, indicating that potassium concentration exerted a more substantial effect on CAT activity (Fig. 2a).

The peroxidase (POD) activities at 40 days post-treatment were significantly higher than those at 20 days post-treatment. At 20 days post-treatment, when Na₂SeO₄ concentration was 1.0 mg·L⁻¹, the highest POD activities were observed at KCl concentrations of 400 mg·L⁻¹ and 100 mg·L⁻¹, with values of 3,220.09 U/g and 3,002.51 U/g, respectively. These values represented 1.77-fold and 1.33-fold increases compared to those at 0 mg·L⁻¹ Na₂SeO₄, indicating that selenium concentration had a more pronounced effect on POD activity. At 40 days post-treatment, when KCl concentration was 100 mg·L⁻¹ and Na₂SeO₄ concentration was 1 mg·L⁻¹, the highest POD activity was 5,571.56 U/g (Fig. 2b).

Superoxide dismutase (SOD) activities at 40 days post-treatment were greater than those at 20 days post-treatment. At 40 days post-treatment, with a constant KCl concentration, varying Na₂SeO₄ concentrations significantly influenced SOD activity, demonstrating that selenium concentration had a more prominent effect on SOD activity. When KCl concentration was 100 mg·L⁻¹ and Na₂SeO₄ concentration was 1.0 mg·L⁻¹, the highest SOD activity was 420.96 U/g, which was 1.47-fold higher than the activity at 0 mg·L⁻¹ Na₂SeO₄ (Fig. 2c).

#### Effects of selenium-potassium combined application on soluble sugar and soluble protein contents in banana leaves

**Fig. 3:**
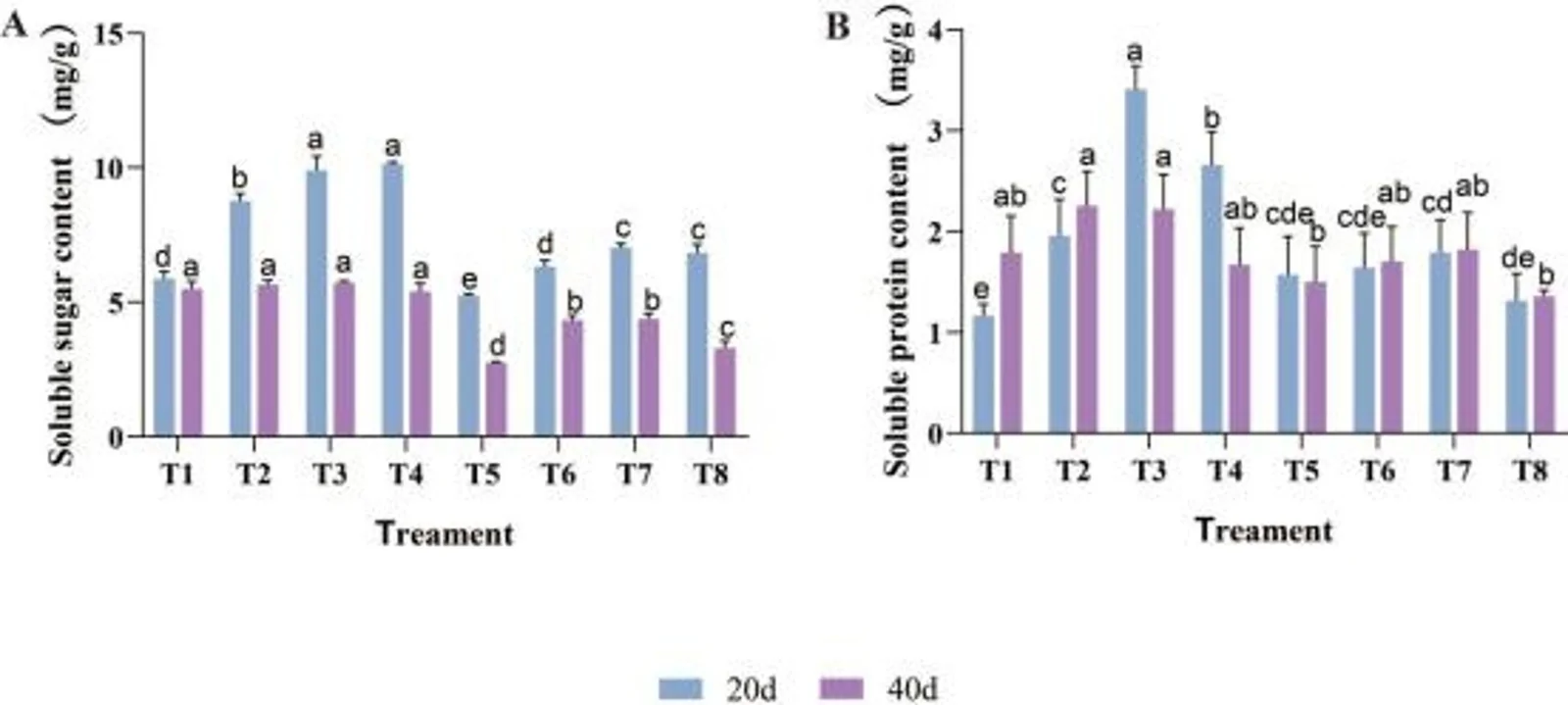
Contents of osmotic adjustment substances in banana leaves under different treatments. **A**, Soluble sugar content; **B**, Soluble protein content

At the same KCl concentration, soluble sugar content was higher at 20 days post-treatment compared to 40 days. When the KCl concentration was 400 mg·L⁻¹, soluble sugar content increased significantly with rising selenium concentrations, reaching a maximum value of 10.13 mg/g at a Na₂SeO₄ concentration of 1.0 mg·L⁻¹. This value was 1.69 times higher than that at 0 mg·L⁻¹ Na₂SeO₄ (Fig. 3a).

When the KCl concentration was 400 mg·L⁻¹, significant differences in soluble protein content were observed among the different selenium concentration treatments. At 20 days post-treatment, the highest soluble protein content of 3.41 mg/g was achieved when the KCl concentration was 400 mg·L⁻¹ and Na₂SeO₄ concentration was 1.0 mg·L⁻¹, which was 2.93 times higher than that at 0 mg·L⁻¹ Na₂SeO₄ (Fig. 3b).

#### Effects of selenium-potassium combined application on selenium and potassium contents in different parts of banana

**Fig. 4:**
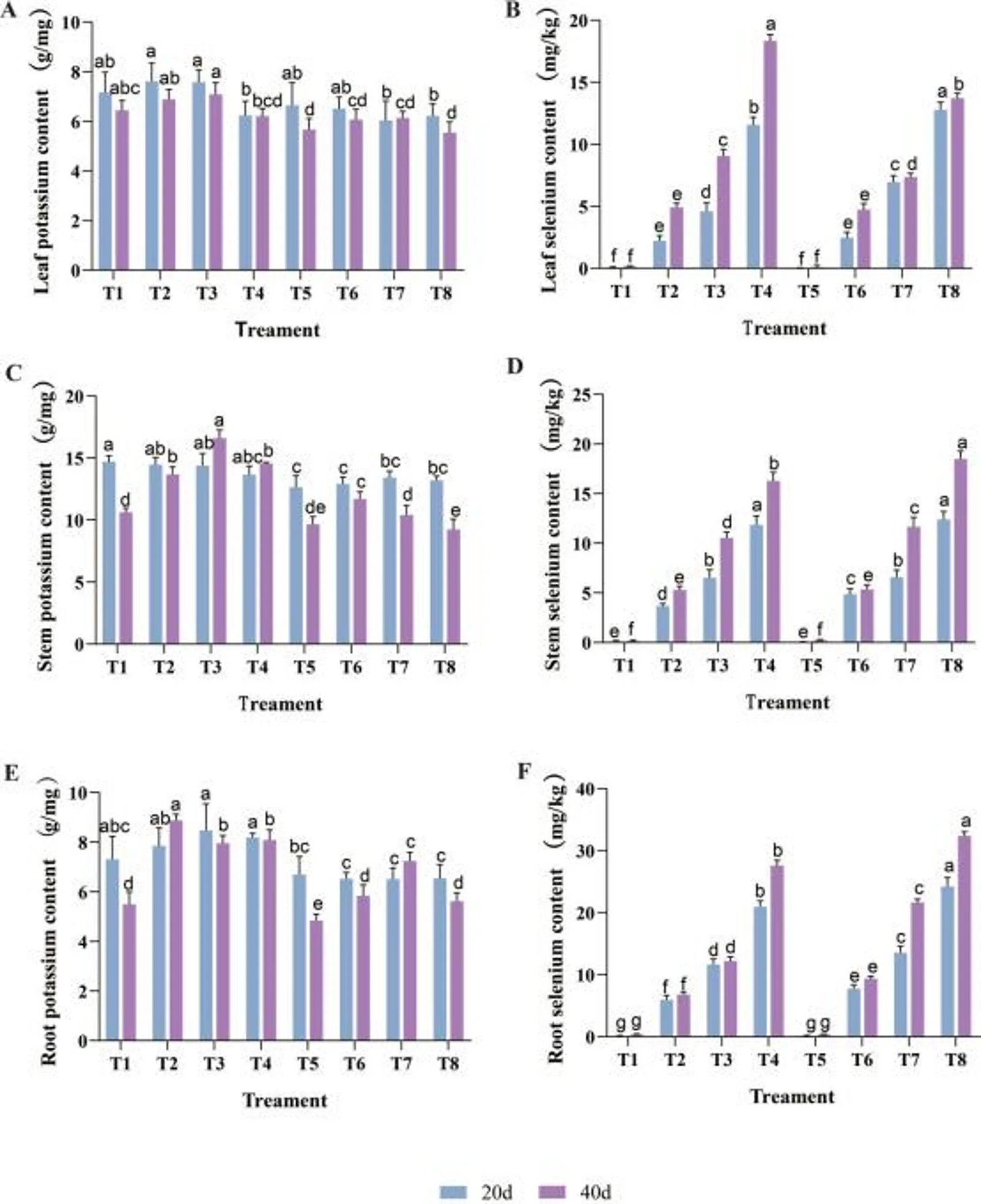
Potassium and selenium contents in leaves, stems and roots of banana seedlings under different treatments. **A**, Potassium content in leaves; **B**, Selenium content in leaves; **C**, Potassium content in stems; **D**, Selenium content in stems; **E**, Potassium content in roots; **F**, Selenium content in roots

The potassium contents in the roots, stems, and leaves of banana under normal potassium concentration treatment were higher than those under low potassium treatment. At 40 days post-treatment, when the KCl concentration was 400 mg·L⁻¹, significant differences were observed in stem potassium content across varying selenium concentrations. The highest stem potassium content of 16.61 g·100 g⁻¹ was recorded at a Na₂SeO₄ concentration of 1.0 mg·L⁻¹, which was 1.56 times greater than that at 0 mg·L⁻¹ Na₂SeO₄. At 40 days post-treatment, when the KCl concentration remained constant, significant differences in root potassium content were noted across varying selenium concentrations. The highest root potassium content of 8.09 g·100 g⁻¹ was achieved at a Na₂SeO₄ concentration of 0.5 mg·L⁻¹, which was 1.62 times higher than at 0 mg·L⁻¹ Na₂SeO₄. When the KCl concentration was 100 mg·L⁻¹ and the Na₂SeO₄ concentration was 1.0 mg·L⁻¹, the highest root potassium content of 7.24 g·100 g⁻¹ was obtained, which was 1.50 times as those at 0 mg·L⁻¹ Na₂SeO₄ (Fig. 4a, c and e), indicating that roots were more sensitive to potassium.

The selenium contents in the roots, stems, and leaves of banana at 40 days post-treatment were higher than those at 20 days post-treatment. When the KCl concentration remained constant, the selenium contents in the roots, stems, and leaves of banana increased significantly with increasing selenium concentrations. At 20 days post-treatment, when the KCl concentration was 400 mg·L⁻¹ and Na₂SeO₄ concentration was 1.0 mg·L⁻¹, the selenium contents in the roots, stems, and leaves were 11.64 mg·kg⁻¹, 6.50 mg·kg⁻¹, and 4.61 mg·kg⁻¹, respectively, which were 87.54, 74.96, and 54.24 times those at 0 mg·L⁻¹ Na₂SeO₄. At 40 days post-treatment, when the KCl concentration was 400 mg·L⁻¹ and Na₂SeO₄ concentration was 1.0 mg·L⁻¹, the selenium contents in the roots, stems, and leaves were 12.21 mg·kg⁻¹, 10.51 mg·kg⁻¹, and 9.06 mg·kg⁻¹, respectively, which were 46.95, 42.04, and 45.3 times as those at 0 mg·L⁻¹ Na₂SeO₄ (Fig. 4b, d and f), indicating that roots were more sensitive to selenium.

### Transcriptome analysis of banana under different selenium and potassium treatments

#### Quality analysis of sequencing data

To investigate the effects of selenium-potassium interactions at different concentrations on the quality and characteristics of banana, transcriptome sequencing was conducted on newly emerged tender leaves and root systems of banana under low potassium treatment with Na₂SeO₄ concentrations of 0 and 1.0 mg·L⁻¹. Quality assessments of the sequencing data were performed for each sample, yielding a total of 181.57 Gb of clean data. The clean data for each sample exceeded 6.38 Gb, with Q20 base percentages surpassing 97.43%, Q30 base percentages above 95.3%, and Guanine-Cytosine content ranging from 50.25% to 53.07% (Supplementary Table 1). These results demonstrate that the sequencing data quality is reliable and satisfies the requirements for subsequent analyses.

#### Analysis of Differentially Expressed Genes (DEGs)

DESeq2 was utilized to perform differential expression analysis on samples from various treatments. DEGs were analyzed between each pair of groups (YT1_vs_YCK1, YT2_vs_YCK2, YCK1_vs_YCK2, YT1_vs_YT2, GT1_vs_GCK1, GT2_vs_GCK2, GCK1_vs_GCK2, GT1_vs_GT2) (Table 6). A total of 2,455 DEGs were identified between YT1 and YCK1 (870 up-regulated and 1,585 down-regulated); 3,530 DEGs were detected between YT2 and YCK2 (2,375 up-regulated and 1,155 down-regulated); 3,134 DEGs were found between YCK1 and YCK2 (2,100 up-regulated and 1,034 down-regulated); 2,541 DEGs were observed between YT1 and YT2 (802 up-regulated and 1,739 down-regulated); 3,422 DEGs were identified between GT1 and GCK1 (1,898 up-regulated and 1,524 down-regulated); 1,101 DEGs were detected between GT2 and GCK2 (1,007 up-regulated and 94 down-regulated); 471 DEGs were found between GCK1 and GCK2 (373 up-regulated and 98 down-regulated); and 3,221 DEGs were observed between GT1 and GT2 (1,765 up-regulated and 1,456 down-regulated).

**Table 6:** Differentially Expressed Genes

Diff Group	Total DEG	up	down
YT1_vs_YCK1	2455	870	1585
YT2_vs_YCK2	3530	2375	1155
YCK1_vs_YCK2	3134	2100	1034
YT1_vs_YT2	2541	802	1739
GT1_vs_GCK1	3422	1898	1524
GT2_vs_GCK2	1101	1007	94
GCK1_vs_GCK2	471	373	98
GT1_vs_GT2	3221	1765	1456

#### Gene Ontology (GO) enrichment analysis of DEGs

**Fig. 5:**
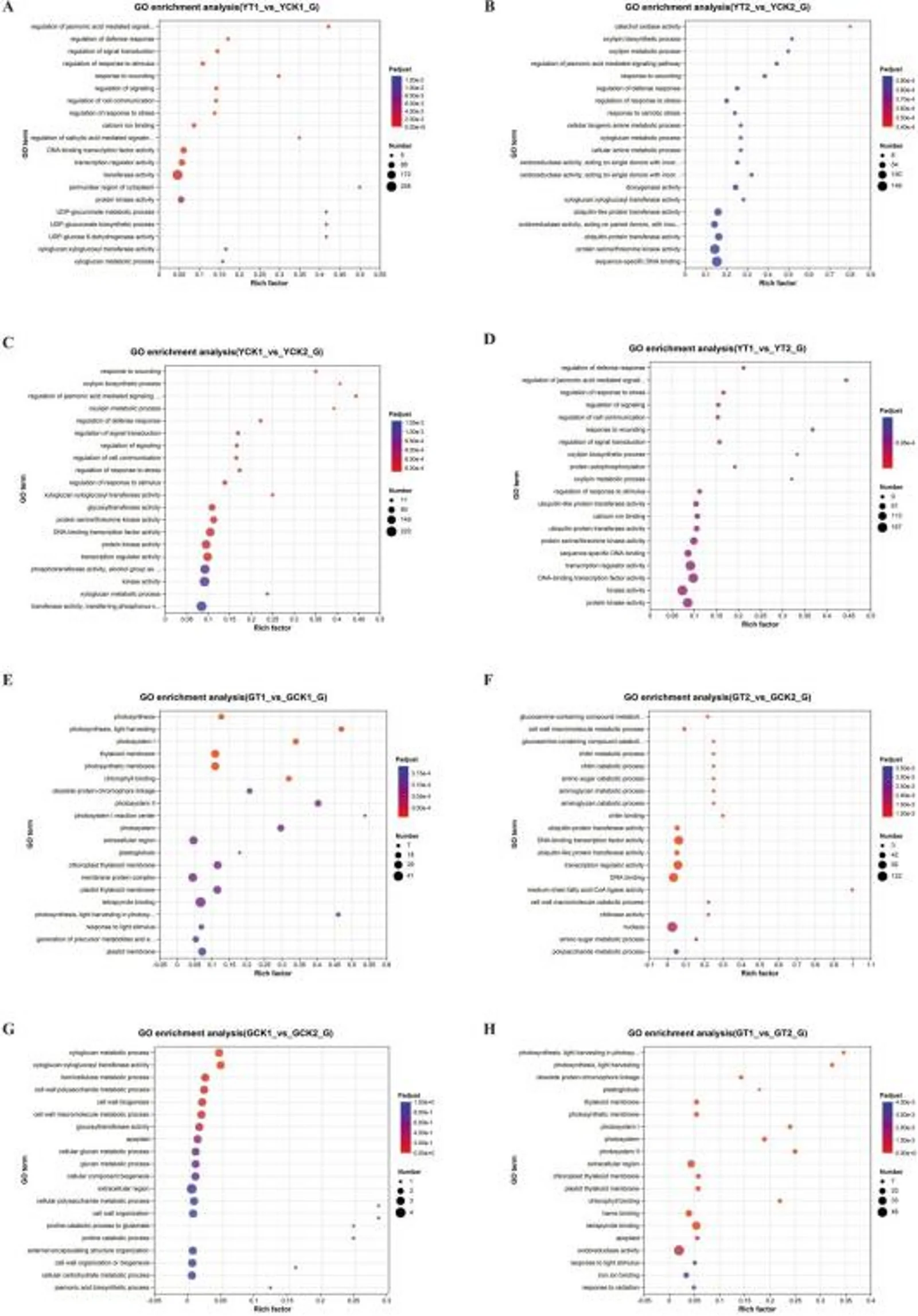
GO Enrichment Analysis

The results of the GO enrichment analysis for eight comparison groups (YT1_vs_YCK1, YT2_vs_YCK2, YCK1_vs_YCK2, YT1_vs_YT2, GT1_vs_GCK1, GT2_vs_GCK2, GCK1_vs_GCK2, and GT1_vs_GT2) are presented in Fig. 5. Based on GO functional annotation, DEGs were significantly (*P* < 0.05) enriched in three major categories: Biological process (BP), cellular component (CC), and molecular function (MF). BP: “Regulation of response to stimulus” and “regulation of cell communication” were prominently enriched across multiple sample groups. In the YT1_vs_YCK1 and YT2_vs_YCK2 groups, this pattern suggests differences in gene regulation related to the detection of external signals and subsequent responses, as well as intercellular communication, which may influence plant adaptation to environmental changes and internal physiological coordination. The “oxylipin metabolic process” was enriched in the YT1_vs_YT2 and YCK1_vs_YCK2 groups. Since oxylipins are involved in plant defense and growth regulation, this suggests potential differences in defense mechanisms and growth regulation between these sample groups, such as variations in disease resistance and developmental progression. MF: “Transferase activity” was identified in multiple groups. Transferases are involved in a variety of biochemical reactions, and their enrichment indicates differences in molecular modification steps of metabolism and signal transduction, such as the synthesis of metabolites and activation of signaling molecules via group transfer. “DNA-binding transcription factor activity” and “protein kinase activity” were recurrently observed. The former regulates gene transcription, while the latter modulates protein function through phosphorylation. These findings suggest differences in key nodes of gene expression regulation and cellular signal transduction pathways, ultimately altering cellular physiological activities and phenotypes. CC: Enrichment related to “photosynthesis, light harvesting” cellular components was observed in the GT1_vs_GCK1 and GT2_vs_GCK2 groups, indicating differences in chloroplast thylakoid membrane-related structures and functions between these two sample groups. These differences likely affect photosynthetic efficiency and reflect variations in light energy utilization and energy conversion. “Perinuclear region of cytoplasm” was represented in the YT1_vs_YCK1 group. As the perinuclear region is involved in nucleocytoplasmic material transport and signal transmission, differential expression of related genes may disrupt communication between the nucleus and cytoplasm, potentially destabilizing intracellular environments and gene expression regulation.

#### Kyoto Encyclopedia of Genes and Genomes (KEGG) enrichment analysis of DEGs

**Fig. 6:**
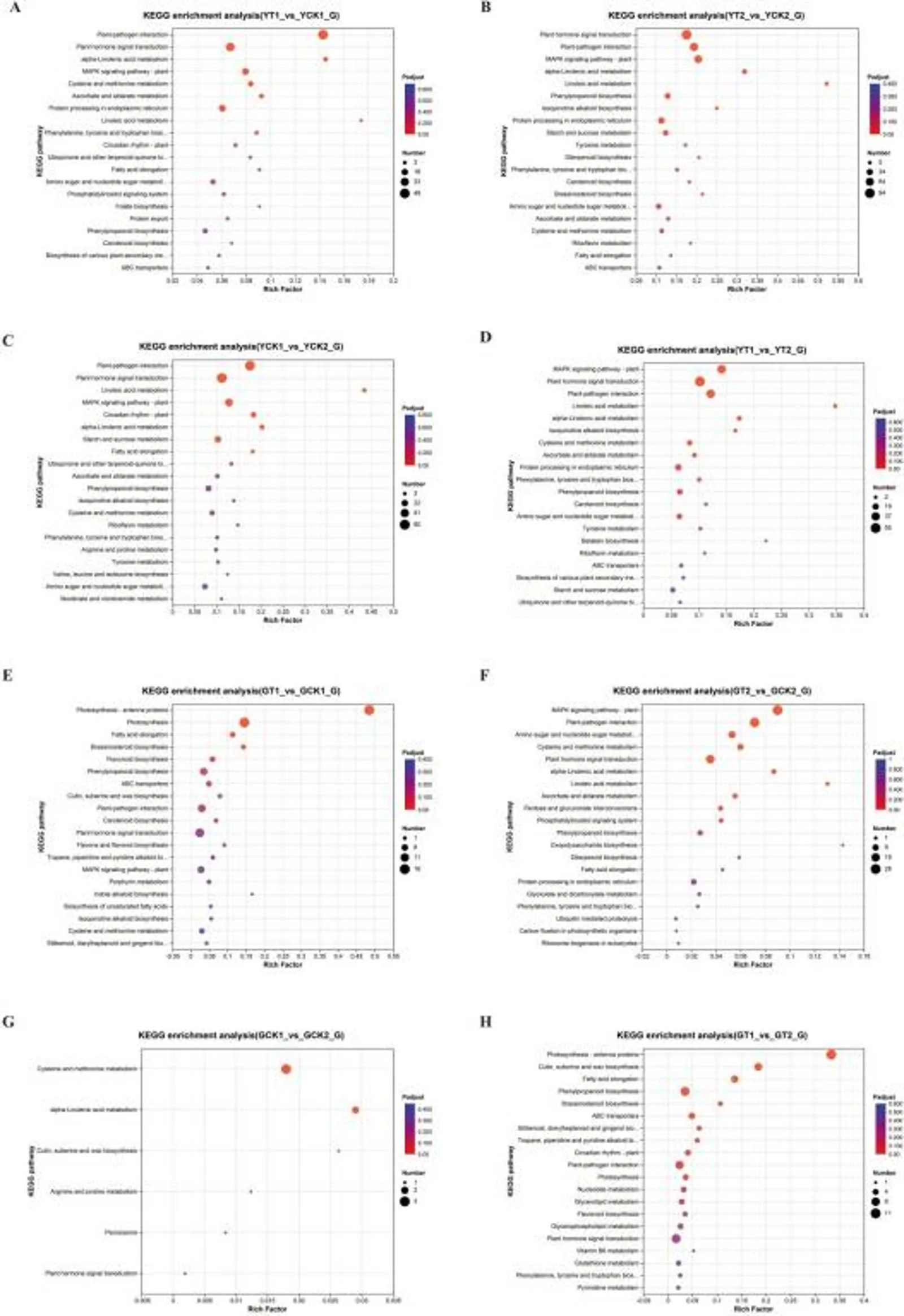
KEGG Enrichment Analysis

The KEGG enrichment analysis results for DEGs from the eight comparison groups (YT1_vs_YCK1, YT2_vs_YCK2, YCK1_vs_YCK2, YT1_vs_YT2, GT1_vs_GCK1, GT2_vs_GCK2, GCK1_vs_GCK2, and GT1_vs_GT2) are shown in Fig. 6. In the YT1_vs_YCK1 group, the top three enriched KEGG pathways were plant-pathogen interaction, plant hormone signal transduction, and protein processing in the endoplasmic reticulum. In the YT2_vs_YCK2, YCK1_vs_YCK2, and YT1_vs_YT2 groups, the most enriched KEGG pathways included plant hormone signal transduction, plant-pathogen interaction, and the MAPK signaling pathway. In the GT1_vs_GCK1 group, the top three enriched KEGG pathways were photosynthesis-antenna proteins, photosynthesis, and plant hormone signal transduction. In the GT2_vs_GCK2 group, the primary enriched KEGG pathways were the MAPK signaling pathway, plant-pathogen interaction, and plant hormone signal transduction. In the GCK1_vs_GCK2 group, the leading enriched KEGG pathways were cysteine and methionine metabolism, alpha-linolenic acid metabolism, and plant hormone signal transduction. In the GT1_vs_GT2 group, the top three enriched KEGG pathways were photosynthesis-antenna proteins, phenylpropanoid biosynthesis, and plant hormone signal transduction. Upon analysis, differences in enrichment results across the comparison groups were observed. Notably, the plant hormone signal transduction pathway was prominently ranked in most groups, highlighting its significant role in gene functional differences across samples. Other pathways, such as plant-pathogen interaction, MAPK signaling pathway, and photosynthesis-related pathways, also emerged as prominent in the enrichment results of various groups, reflecting differential gene functional changes related to growth and development, defense responses, and metabolic processes across the samples.

#### Analysis of plant hormone signal transduction pathways

**Fig. 7:**
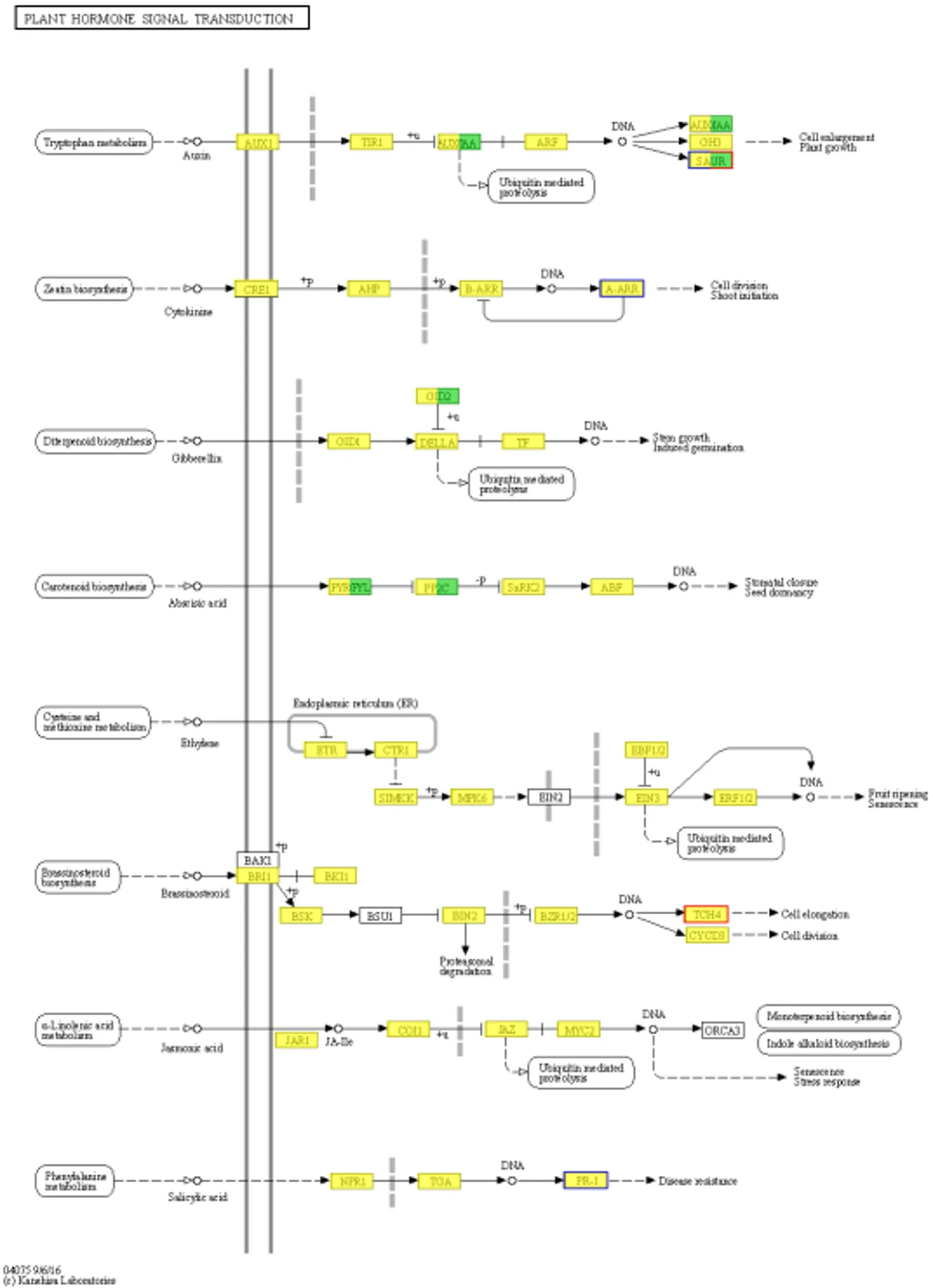
Plant Hormone Signal Transduction Pathway

**Fig. 8:**
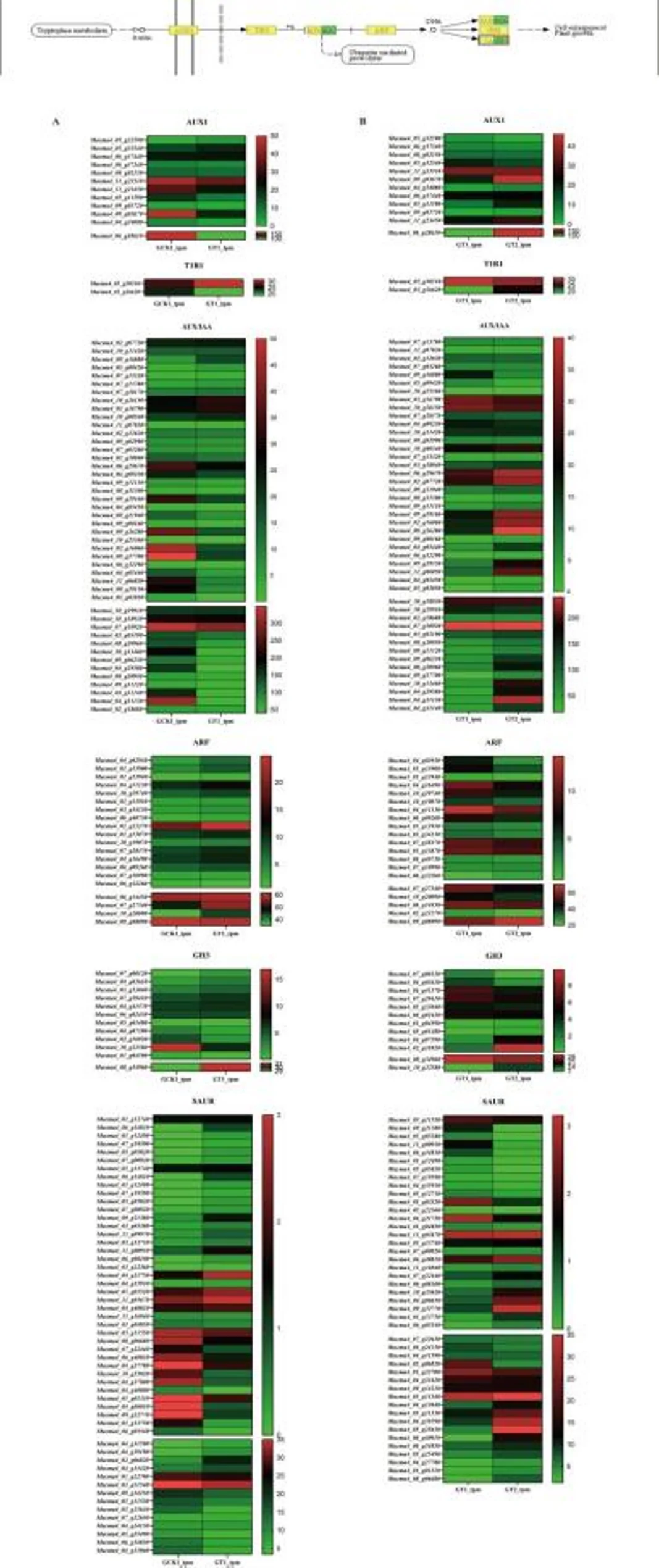
Auxin Signal Transduction Pathway. **A**, GT1 vs. GCK1; **B**, GT1 vs. GT2

**Fig. 9:**
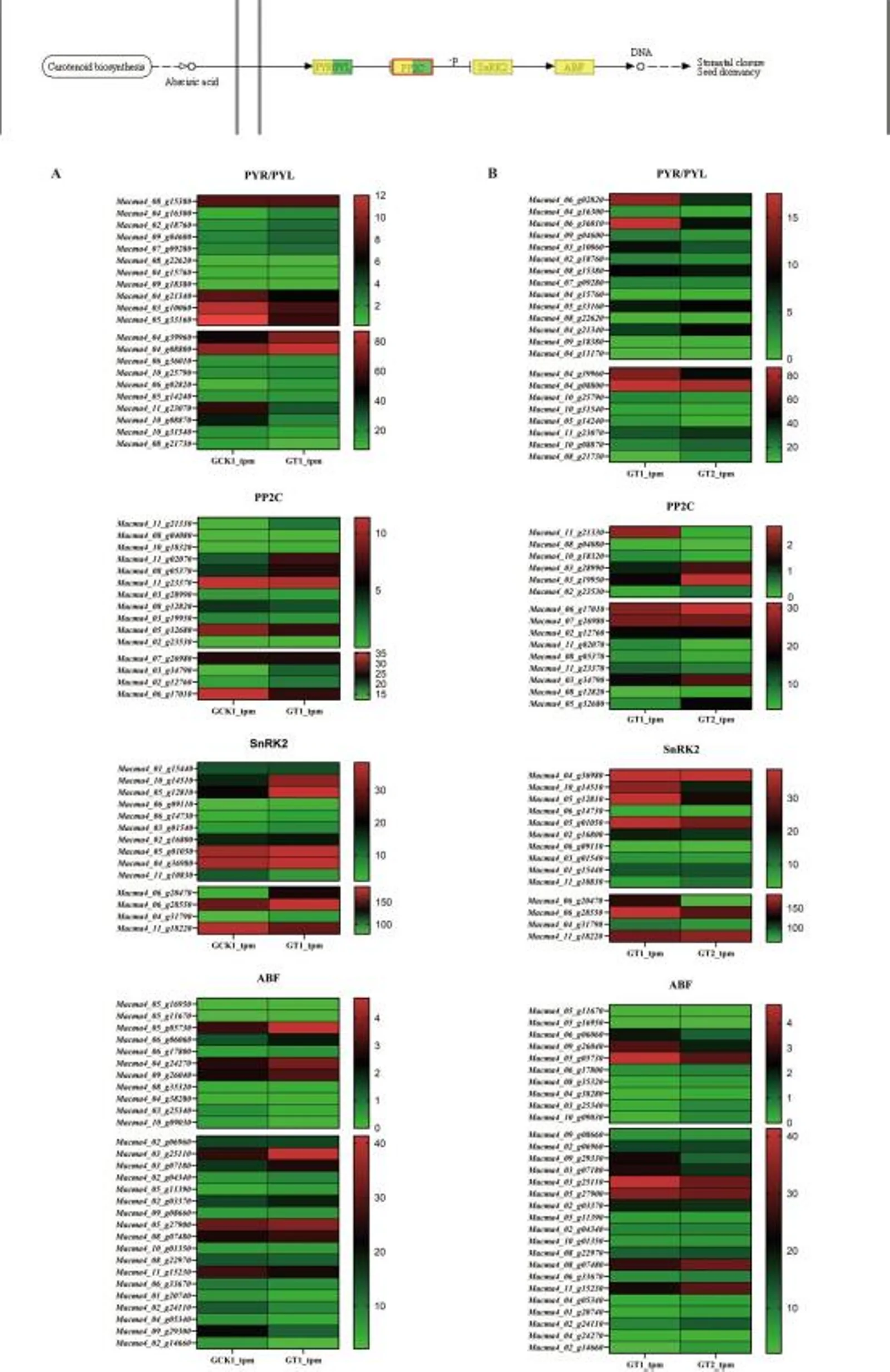
Abscisic Acid Signal Transduction Pathway. **A**, GT1 vs. GCK1; **B**, GT1 vs. GT2

This study confirmed that the root system of banana seedlings exhibits higher sensitivity to combined selenium and potassium application at different concentrations. KEGG pathway analysis revealed significant enrichment of DEGs in plant hormone signal transduction pathways. Therefore, further analysis was conducted on the plant hormone signal transduction pathway (Fig. 7) for Group A (GT1_vs_GT2, with Na₂SeO₄ concentration uniformly at 1.0 mg·L⁻¹, KCl concentration at 400 mg·L⁻¹ in GT1 and 100 mg·L⁻¹ in GT2) and Group B (GT1_vs_GCK1, with KCl concentration uniformly at 400 mg·L⁻¹, Na₂SeO₄ concentration at 1.0 mg·L⁻¹ in GT1 and 0 mg·L⁻¹ in GCK1). In Group A, 472 DEGs were identified, comprising 277 up-regulated and 195 down-regulated genes. In Group B, 470 DEGs were detected, including 273 up-regulated and 197 down-regulated genes (Supplementary Table 2). Furthermore, considerable DEG enrichment was observed in both auxin signal transduction (Fig. 8) and abscisic acid (ABA) signal transduction (Fig. 9) pathways across Groups A and B.

##### Auxin signal transduction pathway

In Group A, within the AUX1 gene family, five genes, including *Macma4_08_g02150* and *Macma4_06_g37340*, were up-regulated, while seven genes, including *Macma4_09_g03670* and *Macma4_04_g24080*, were down-regulated, indicating significant alterations in AUX1-mediated auxin uptake or transport processes under the various treatments. For example, the upregulation of *Macma4_05_g32590* likely enhanced auxin absorption, promoting plant cell elongation, division, and differentiation. The TIR1 gene *Macma4_05_g30510* was up-regulated, whereas *Macma4_03_g26620* was down-regulated, affecting the initiation of auxin signaling. Within the AUX/IAA gene family, multiple members displayed marked expression differences, with 13 genes, including *Macma4_07_g13780*, up-regulated and 28 genes, including *Macma4_02_g18680*, down-regulated. As repressors in the auxin signaling pathway, the complex expression patterns of AUX/IAA proteins suggest the existence of intricate feedback regulatory mechanisms within this pathway. In Group B, AUX1 genes *Macma4_05_g32590* and *Macma4_05_g32540* were up-regulated, while 10 genes, including *Macma4_06_g37440*, were down-regulated. Compared to Group A, the differences in the quantity and types of DEGs reflect distinct effects of the various treatments on auxin uptake and transport.

##### Cytokinin signal transduction pathway

In Group A, 10 CRE1 genes, including *Macma4_04_g18360* and *Macma4_07_g11220*, were up-regulated, while *Macma4_09_g20280* was down-regulated, indicating differential cytokinin signal perception between the two groups, which may influence the initiation of cytokinin signaling. Six AHP genes, including *Macma4_02_g04460* and *Macma4_09_g16440*, were up-regulated, while three genes, including *Macma4_11_g24500* and *Macma4_11_g25420*, were down-regulated, participating in the regulation of cytokinin signal transduction from receptors to the nucleus. In Group B, eight CRE1 genes, including *Macma4_09_g20270* and *Macma4_04_g18360*, were up-regulated, while *Macma4_11_g12000* and *Macma4_09_g20280* were down-regulated. Although some genes exhibited similar trends compared to Group A, overall expression differences were evident, suggesting that the different selenium-potassium concentration treatments differentially affected cytokinin signal perception in banana seedling roots, thereby influencing plant cell division, differentiation, and tissue-organ development.

##### Gibberellin signal transduction pathway

In Group A, five GID1 genes, including *Macma4_06_g17910* and *Macma4_10_g16510*, were up-regulated, while *Macma4_08_g05590* was down-regulated, impacting gibberellin-receptor binding and signal initiation. DELLA genes, such as *Macma4_08_g18720* and *Macma4_01_g15240*, were up-regulated, while four genes, including *Macma4_02_g26000* and *Macma4_10_g23680*, were down-regulated. As negative regulators, changes in their expression altered gibberellin responses, influencing processes like internode elongation. In Group B, five GID1 genes, including *Macma4_03_g27620* and *Macma4_10_g16510*, were up-regulated, while *Macma4_08_g05590* was down-regulated. Similar to Group A, these results suggest comparable effects of different selenium-potassium concentration treatments on gibberellin signal initiation in banana seedlings. However, subsequent physiological processes may differ due to alterations in other genes.

##### ABA signal transduction pathway

In Group A, 13 PYR/PYL genes, including *Macma4_06_g02820* and *Macma4_06_g36010*, were up-regulated, while nine genes, including *Macma4_04_g15760* and *Macma4_05_g33160*, were down-regulated, influencing ABA signal initiation. Nine PP2C genes, including *Macma4_06_g17010* and *Macma4_07_g26980*, were up-regulated, while six genes, including *Macma4_03_g34790* and *Macma4_08_g12820*, were down-regulated. As key regulators in signal transduction, changes in their expression altered both the intensity and direction of signal transmission. In Group B, 10 PYR/PYL genes, including *Macma4_08_g15380* and *Macma4_04_g16300*, were up-regulated, while 11 genes, including *Macma4_05_g14240* and *Macma4_08_g22620*, were down-regulated. Compared to Group A, the differences in gene expression reflected distinct effects of varying selenium-potassium concentration treatments on ABA signal initiation and transmission in banana seedlings, thereby influencing physiological processes such as stress responses and growth and development.

##### Ethylene signal transduction pathway

Six ETR genes, including *Macma4_11_g17530* and *Macma4_08_g12840*, were up-regulated in both Groups A and B, while *Macma4_06_g37000* and *Macma4_03_g14820* were down-regulated, affecting ethylene signal perception. In Group A, 12 EIN3 genes, including *Macma4_08_g27120* and *Macma4_10_g34110*, were up-regulated, while three genes, including *Macma4_08_g22960* and *Macma4_03_g09530*, were down-regulated. As transcription factors, they regulated ethylene-responsive gene expression, influencing processes such as fruit ripening, senescence, and stress responses. In Group B, 14 EIN3 genes, including *Macma4_07_g24540* and *Macma4_08_g22960*, were up-regulated, while two genes, including *Macma4_06_g00890*, were down-regulated. Although some genes exhibited consistent changes with Group A, the overall signaling pathway displayed distinct regulatory mechanisms due to differences in other genes.

##### Brassinosteroid signal transduction pathway

BRI1 genes (*Macma4_01_g26960*, *Macma4_04_g09190*, *Macma4_06_g39530*) were down-regulated in both Groups A and B, affecting brassinosteroid signal initiation. In Group A, four BKI1 genes, including *Macma4_07_g13030*, were up-regulated, regulating BRI1 activity. In Group B, three BKI1 genes, including *Macma4_05_g26400*, were up-regulated, while *Macma4_07_g13030* was down-regulated. Gene expression differences under varying selenium-potassium concentrations influenced brassinosteroid signal transduction, thereby affecting processes such as plant cell elongation, division, and photomorphogenesis.

##### Jasmonic acid signal transduction pathway

In Group A, four JAR-1 genes, including *Macma4_04_g16260* and *Macma4_07_g27610*, were up-regulated, while *Macma4_03_g34760* was down-regulated, participating in jasmonic acid synthesis and affecting its content. Twelve JAZ genes, including *Macma4_04_g09960* and *Macma4_06_g34370*, were up-regulated, while 19 genes, including *Macma4_07_g22660* and *Macma4_08_g24380*, were down-regulated, acting as repressors to regulate jasmonic acid signal transduction. In Group B, four JAR-1 genes, including *Macma4_04_g16260* and *Macma4_07_g27610*, were up-regulated, four COI1 genes, including *Macma4_09_g03770* and *Macma4_05_g32610*, were up-regulated, and 12 JAZ genes, including *Macma4_10_g35050* and *Macma4_03_g01960*, were up-regulated, while 14 genes, including *Macma4_04_g09960* and *Macma4_09_g15840*, were down-regulated. Gene expression differences between the two groups influenced jasmonic acid signal transduction, playing pivotal roles in plant defense responses as well as growth and development processes.

##### Salicylic acid signal transduction pathway

In Group A, 10 NPR1 genes, including *Macma4_10_g06240* and *Macma4_05_g08300*, were up-regulated, while five genes, including *Macma4_11_g19640* and *Macma4_08_g05180*, were down-regulated. As key regulators, these genes influenced salicylic acid signal transmission and downstream defense gene expression. In Group B, eight NPR1 genes, including *Macma4_06_g02790* and *Macma4_10_g06240*, were up-regulated, while six genes, including *Macma4_07_g09160* and *Macma4_03_g28590*, were down-regulated. Compared to Group A, gene expression differences affected physiological processes, such as salicylic acid-mediated plant disease resistance.

#### Validation of differential gene expression by quantitative real-time polymerase chain reaction (qRT-PCR)

**Fig. 10:**
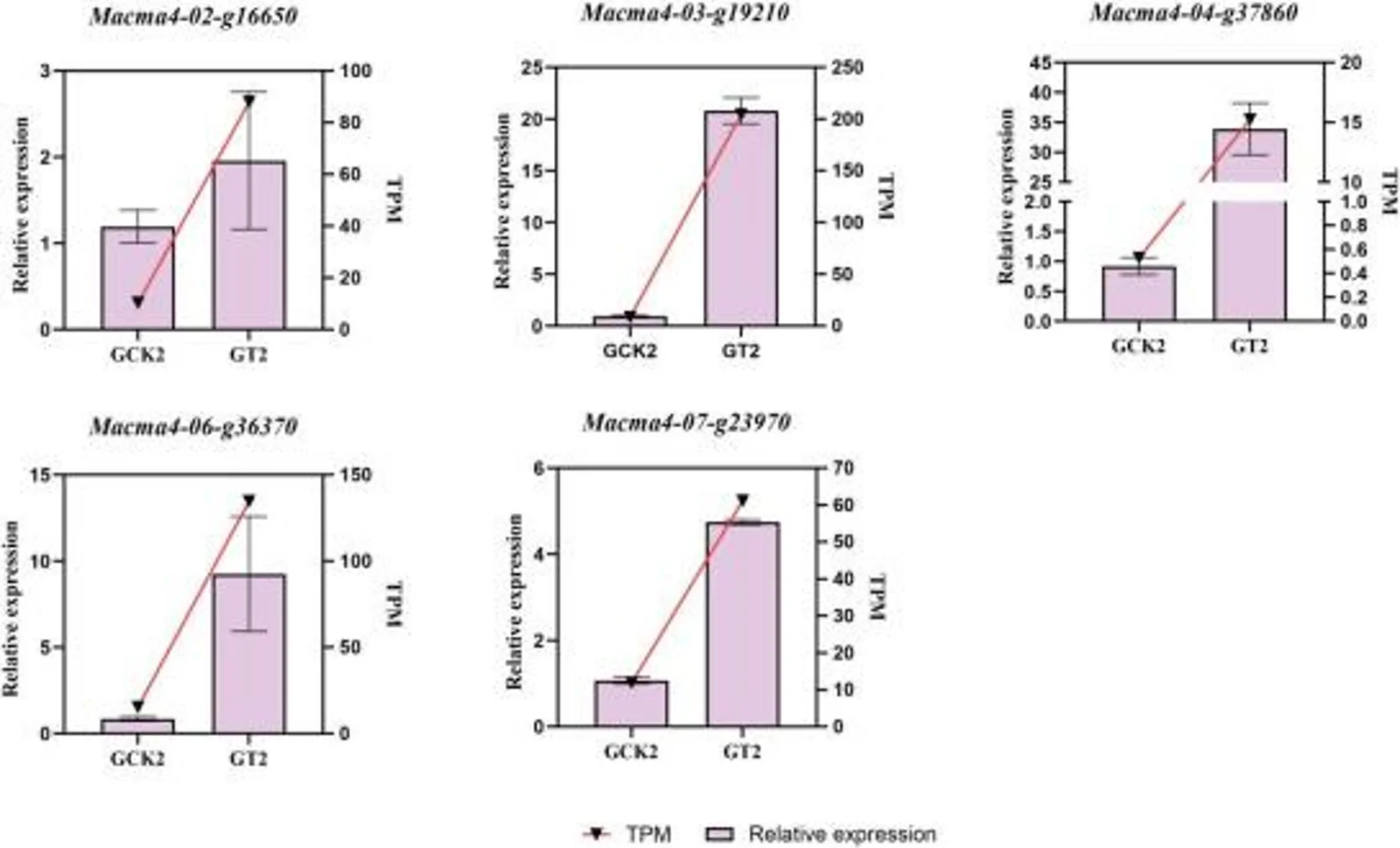
Real-Time Fluorescent Quantitative PCR

To validate the accuracy of the transcriptome results, five DEGs from T5 and T7 root samples were randomly selected for further qRT-PCR analysis. The overall differential expression trends were found to be largely consistent, confirming that the transcriptome data were accurate and reliable (Fig. 10).

## Discussion

### Effects of combined application of selenium-potassium on physiological indices of banana seedlings

Appropriate concentrations of selenium and potassium promote the growth and development of banana. In this study, after 40 days of treatment under normal potassium concentration, selenium concentration showed no significant effect on the fresh and dry weights of roots, stems, and leaves, indicating that the mass of different parts of banana had reached peak values and was unaffected by selenium content. After 20 days of treatment, under both normal and low potassium concentrations, the mass of different parts of banana was highest when Na₂SeO₄ concentration was 1.0 mg·L⁻¹, with a higher mass observed under normal potassium content. This suggests that appropriate concentrations of selenium and potassium promote the growth of roots, stems, and leaves in banana. The trends in the accumulation of soluble sugar and soluble protein content were consistent with the changes in mass, indicating that a KCl concentration of 400 mg·L⁻¹ and Na₂SeO₄ concentration of 1.0 mg·L⁻¹ represent the optimal concentrations. In the leaves of banana seedlings, compared to low potassium treatment (YCK1_vs_YCK2), normal potassium concentration treatment resulted in significant upregulation of sucrose-phosphate synthase gene (*Macma4_06_g19230*), hexokinase gene (*Macma4_03_g34440*), fructokinase gene (*Macma4_07_g13720*) and others ( Supplementary Table 3. The functional associations of these genes are derived from homology-based annotation predictions using transcriptomic data, rather than experimentally validated phenotypes. Independent molecular functional verification is required in future investigations, and this principle applies to subsequent genes mentioned hereinafter ), thereby increasing carbohydrate synthesis in the leaves, which were then transported to utilization sites in the plant, leading to increased plant mass. Meanwhile, potassium facilitates the division of living plant cells, promotes the growth of meristematic tissues, and aids in carbohydrate formation and the translocation of substances produced during photosynthesis [40, 41]. In this study, potassium treatment enhanced the expression levels of photosystem II (*Macma4_06_g31350*), photosystem I (*Macma4_07_g13100*), cytochrome b6/f complex (*Macma4_09_g26540*) and others ( Supplementary Table 3 ), thereby increasing photosynthetic output and promoting the accumulation of plant nutrients and dry weight. Selenium enhances plant chlorophyll content, improves crop photosynthesis, elevates antioxidant enzyme activity, strengthens antioxidant capacity, and enhances plant stress resistance and anti-aging ability, thereby promoting plant growth and dry matter accumulation [42, 43].

### Effects of combined application of selenium-potassium on antioxidant enzymes in banana leaves

When plants are subjected to stress, the activities of protective enzymes within the plant change accordingly. SOD, POD, and CAT are essential enzymes involved in scavenging excess O2^•−^, H₂O₂, and other reactive oxygen species (ROS) in plants. These enzymes play critical roles in resisting various physicochemical stress factors, reducing ROS accumulation, and maintaining membrane integrity, thereby enabling plants to tolerate, alleviate, or resist stress-induced damage to a certain extent [44, 45]. Selenium participates in the formation of the antioxidant defense system in plants. When the Na₂SeO₄ concentration was 1.0 mg·L⁻¹, compared to no selenium application (YT1_vs_YCK1), genes encoding POD (*Macma4_08_g12030*), SOD (*Macma4_02_g05820*), and CAT (*Macma4_01_g11370*) in banana seedling leaves were significantly up-regulated ( Supplementary Table 3 ), leading to enhanced activities of antioxidant enzymes such as SOD, POD, and CAT. This increase in enzyme activities aids in scavenging free radicals generated during plant growth. These findings align with the results of Nawaz et al. [46]. Potassium regulates the osmotic potential of plant cells and enhances their water retention capacity, enabling plants to better cope with adverse conditions such as drought and high temperatures [47]. Under combined selenium and potassium application, when banana seedlings encounter unfavorable environmental conditions, such as drought stress, the decrease in relative water content in the leaves is smaller compared to seedlings without combined application, maintaining better growth, reducing leaf yellowing and growth stagnation caused by stress, and improving the survival rate and vigor of the seedlings [48]. In this study, exogenous application of selenium and potassium significantly enhanced the activities of protective enzymes in banana leaves, particularly when KCl concentration was 400 mg·L⁻¹ and Na₂SeO₄ concentration was 1.0 mg·L⁻¹. Under these conditions, enzyme activities were relatively high, improving the adaptability of banana to environmental stress and increasing yield.

### Effects of combined application of selenium-potassium on selenium and potassium accumulation in roots, stems, and leaves of banana

The roots and stems of banana at the seedling stage exhibit a higher demand for potassium [49], which aligns with the findings of the present study. When various concentrations of KCl were applied, no significant difference in potassium content was observed in the leaves of banana. However, at a KCl concentration of 400 mg·L⁻¹, the potassium content in the roots and stems was significantly higher compared to that at 100 mg·L⁻¹ KCl. Under normal potassium concentration treatment, compared with low potassium treatment (GCK1_vs_GCK2), genes associated with potassium absorption and transport in the roots of banana seedlings, such as calcineurin B-like protein (*Macma4_03_g13610*) and potassium transporter (*Macma4_06_g26820*), were up-regulated ( Supplementary Table 3 ), thereby facilitating potassium accumulation. Liu et al. [34] demonstrated that selenium application at the seedling stage could significantly increase selenium content in banana fruits. In the current study, with a constant potassium concentration, selenium content in the roots, stems, and leaves of banana increased significantly with rising selenium concentrations, with the roots exhibiting greater sensitivity to Na₂SeO₄. Selenium absorption by plant roots primarily occurs through the sulfate assimilation pathway [50]. Under normal potassium concentration, when Na₂SeO₄ concentration was 1.0 mg·L⁻¹, compared to no selenium fertilizer application (GT1_vs_GCK1), genes such as cysteine synthase (*Macma4_02_g19970*), methionine γ-lyase (*Macma4_11_g03450*), and γ-glutamyl transpeptidase (*Macma4_06_g26020*) were significantly upregulated ( Supplementary Table 3 ), promoting selenium accumulation in the roots of banana seedlings. According to DB45/T 1061—2014, the appropriate selenium content for selenium-enriched fruits ranges from 0.01 to 0.10 mg·kg⁻¹. At a Na₂SeO₄ concentration of 1.0 mg·L⁻¹, the selenium content in the roots, stems, and leaves of banana met the selenium-enrichment requirement, with 400 mg·L⁻¹ KCl treatment showing significantly higher selenium content than the 100 mg·L⁻¹ KCl treatment. These results suggest that the combination of 400 mg·L⁻¹ KCl and 1.0 mg·L⁻¹ Na₂SeO₄ facilitates the accumulation of both selenium and potassium in banana.

### Effects of combined application of selenium-potassium on plant hormone signal transduction pathways in banana roots

Transcriptomic analysis was employed in this study to analyze gene expression changes in the roots, stems, and leaves of banana under varying selenium-potassium combined application concentrations. KEGG enrichment analysis revealed significant enrichment in plant hormone signal transduction pathways, irrespective of selenium-potassium application concentrations or tissue types. Further examination of the plant hormone signal transduction pathways in the GT1_vs_GT2 and GT1_vs_GCK1 groups demonstrated that numerous DEGs were enriched in the auxin and ABA signal transduction pathways.

In the auxin signal transduction pathway, when Na₂SeO₄ concentration was maintained at 1.0 mg·L⁻¹, different KCl concentrations influenced the growth and development of banana seedlings. Under normal potassium concentration, the fresh and dry weights of roots, stems, and leaves of banana seedlings were higher compared to those under low potassium treatment, which can be attributed to the impact of gene expression in the auxin signaling pathway on plant growth. Significant differential expression of genes such as AUX1, AUX/IAA, and ARF was observed between low potassium and normal potassium concentration treatments. As an auxin influx carrier, the AUX1 gene was more down-regulated under low potassium treatment, potentially reducing auxin uptake and impairing cell elongation and division, which corresponded to the relatively slower growth of plants under low potassium conditions. Members of the AUX/IAA gene family exhibited complex expression patterns under different potassium concentration treatments, with multiple genes showing both upregulation and downregulation trends. This suggests that varying potassium concentrations finely regulate the auxin signaling pathway by modulating AUX/IAA gene expression, thereby influencing plant growth and developmental processes. WANG [51] demonstrated that auxin, jasmonic acid, ethylene, and other hormones are involved in potassium signal transduction, and under low potassium stress, these hormones enhance the stress resistance of Ipomoea batatas by regulating upstream transcription factors. When the KCl concentration was 400 mg·L⁻¹ and Na₂SeO₄ concentration varied, the growth and development of banana seedlings were similarly influenced through changes in gene expression within the auxin signaling pathway. The AUX1 gene was up-regulated, with genes such as *Macma4_05_g32590* at Na₂SeO₄ concentration of 1.0 mg·L⁻¹, while genes such as *Macma4_06_g37440* were down-regulated at Na₂SeO₄ concentration of 0 mg·L⁻¹, suggesting that selenium supplementation may enhance auxin uptake in banana roots. Several ARF genes, including *Macma4_04_g02930*, were up-regulated at Na₂SeO₄ concentration of 1.0 mg·L⁻¹, while genes such as *Macma4_09_g08090* were down-regulated at Na₂SeO₄ concentration of 0 mg·L⁻¹, indicating that selenium may induce more active auxin response genes in the roots of banana seedlings at 1.0 mg·L⁻¹ Na₂SeO₄, thereby promoting plant growth and reflecting a positive regulatory effect of selenium on the auxin signaling pathway. This finding aligns with YAO’s research [52], which demonstrated, through transcriptome analysis of *Mesembryanthemum crystallinum* under selenium treatment, that increased selenium concentration promoted auxin and gibberellin signal transduction, activating downstream gene expression while also influencing the expression of key genes in the jasmonic acid and salicylic acid signaling pathways, thus regulating selenium-mediated stress responses.

Selenium-potassium combined application enhances the activities of antioxidant enzymes, such as SOD, POD, and CAT, in the leaves of banana seedlings, reducing ROS accumulation and mitigating oxidative damage caused by ROS. However, under moderate redox signal regulation, the ABA signaling pathway is activated, leading to increased ABA content. ABA induction subsequently increases osmotic substances, such as soluble proteins and soluble sugars, in the leaves of banana seedlings, lowering cellular osmotic potential and thereby enhancing plant stress resistance. Within the ABA signal transduction pathway, different KCl concentrations influence the expression of genes such as PYR/PYL, PP2C, and SnRK2. PYR/PYL genes, including *Macma4_06_g02820*, were upregulated at a KCl concentration of 400 mg·L⁻¹, while some PYR/PYL genes were down-regulated under low potassium conditions, potentially impairing the initiation of ABA signaling and weakening the stress response of banana seedlings. Expression differences in PP2C and SnRK2 genes under varying potassium concentrations also suggested that low potassium treatment may disrupt ABA signal transmission and regulatory mechanisms, altering plant sensitivity and response patterns to ABA in a low potassium environment, thus affecting stress adaptation. Research by Zhu et al. [53] demonstrated that potassium deficiency-induced PP2C expression was significantly enhanced, negatively regulating ABA signal transduction and exacerbating the inhibitory effects under drought stress. When the KCl concentration was 400 mg·L⁻¹ and the Na₂SeO₄ concentration varied, the ABA signaling pathway was influenced. PYR/PYL genes, including *Macma4_08_g15380*, were up-regulated at Na₂SeO₄ concentration of 1.0 mg·L⁻¹, whereas genes such as *Macma4_05_g14240* were down-regulated at Na₂SeO₄ concentration of 0 mg·L⁻¹, suggesting that selenium presence may enhance the initiation of ABA signaling in banana roots. Changes in the expression of PP2C and SnRK2 genes further indicated that selenium may modulate ABA signal intensity by regulating these genes, thereby impacting plant stress adaptation. Research by Yao et al. [52] demonstrated that low selenium concentration treatment (10 mmol/L) significantly reduced SnRK2 expression, negatively regulating the downstream transcription factor ABF in ABA signal transduction, inhibiting stomatal closure, and increasing dehydration susceptibility in *Mesembryanthemum crystallinum*. Therefore, selenium treatment at specific concentrations may enhance plant adaptability to stress. In this study, Na₂SeO₄ concentration of 1.0 mg·L⁻¹ may improve banana’s adaptation to environmental changes. The current conclusions are only supported by transcriptomic data at the transcriptional level, while quantification of endogenous hormone contents is lacking. We will conduct systematic quantitative detection of endogenous hormones in follow-up research to refine and validate the regulatory mechanism proposed in this study.

## Conclusions

The selenium-potassium combined application affects the growth and development of banana seedlings. The optimal combination of concentrations is 400 mg·L⁻¹ KCl and 1.0 mg·L⁻¹ Na₂SeO₄, which promotes seedling growth and enhances their adaptability to stress conditions. Transcriptome sequencing analysis revealed that DEGs were significantly enriched in plant hormone signal transduction pathways, providing a basis for further investigation into the hormonal signal transduction pathways involved in the selenium-potassium regulation processes.

## Supplementary Information

Supplementary Material 1.

Supplementary Material 2.

Supplementary Material 3.

## Data Availability

The datasets supporting the conclusions of this study are included in the article and its supplementary materials, or are available from the corresponding author upon reasonable request. The raw transcriptome sequence data generated during the current study are available in the NCBI repository under the BioProject accession number PRJNA1445110.
